# Right sleeve pneumonectomy for local recurrent lung cancer following right sleeve upper lobectomy with bronchoplasty: a case report

**DOI:** 10.1186/s13019-020-01175-2

**Published:** 2020-06-09

**Authors:** Jun Hanaoka, Yo Kawaguchi, Keigo Okamoto, Ryosuke Kaku, Yasuhiko Ohshio

**Affiliations:** Division of General Thoracic Surgery, Department of Surgery, Shiga University of Medical Science, Tsukinowacho, Seta, Otsu, Shiga 520-2192 Japan

**Keywords:** Sleeve pneumonectomy, Salvage surgery, Post sleeve lobectomy, Two-stage approach, Recurrent lung cancer

## Abstract

**Background:**

Salvage surgery has been frequently performed, increasing the opportunity to actively perform surgery for recurrence after a function-preserving operation. However, re-operation after airway reconstruction surgery on the proximal side and the effect of prior treatment, such as radiotherapy and/or chemotherapy, make the operation more difficult. In addition, cases of sleeve pneumonectomy after sleeve lobectomy with bronchoplasty are uncommon.

**Case presentation:**

A 71-year-old lung cancer patient underwent right upper sleeve lobectomy with bronchoplasty combined with perioperative chemotherapy in 2007. A new undiagnosed right hilar mass that appeared 9 years post-operation showed a temporary response to radiotherapy but progressed thereafter. Sleeve pneumonectomy was completed 14 months after radiotherapy by the following procedures: dividing the right pulmonary artery at the proximal site under median sternotomy and then reconstructing the bronchus by telescoping the left main bronchus into the distal trachea after pneumonectomy under posterolateral thoracotomy.

**Conclusions:**

Sleeve pneumonectomy for recurrent lung cancer could be safely performed under good vision using a two-stage approach as salvage surgery, even in high-risk patients who received various treatments and proximal airway reconstruction.

## Background

Radical surgery combined with airway reconstruction helps conserve respiratory function by avoiding pneumonectomy for centrally occurring primary lung cancer. Airway reconstruction surgery as radical resection has comparable local recurrence rate and prognosis to pneumonectomy. If such cases relapse unfortunately, they may be treated initially with radiotherapy and/or chemotherapy, but may become recurrent or refractory. Recently, salvage surgery is performed more frequently if strict indications are met for treatment of these cases [1]. However, for airway reconstruction surgery on the proximal side, the difficulty of securing the airway and vessels more centrally and the effect of prior treatment make the operation more difficult. Herein, we report a case of local recurrent lung cancer after right sleeve upper lobectomy with bronchoplasty and adjuvant chemotherapy, which showed the beneficial effect of right sleeve pneumonectomy at progression after radiotherapy.

## Case presentation

**Fig. 1:**
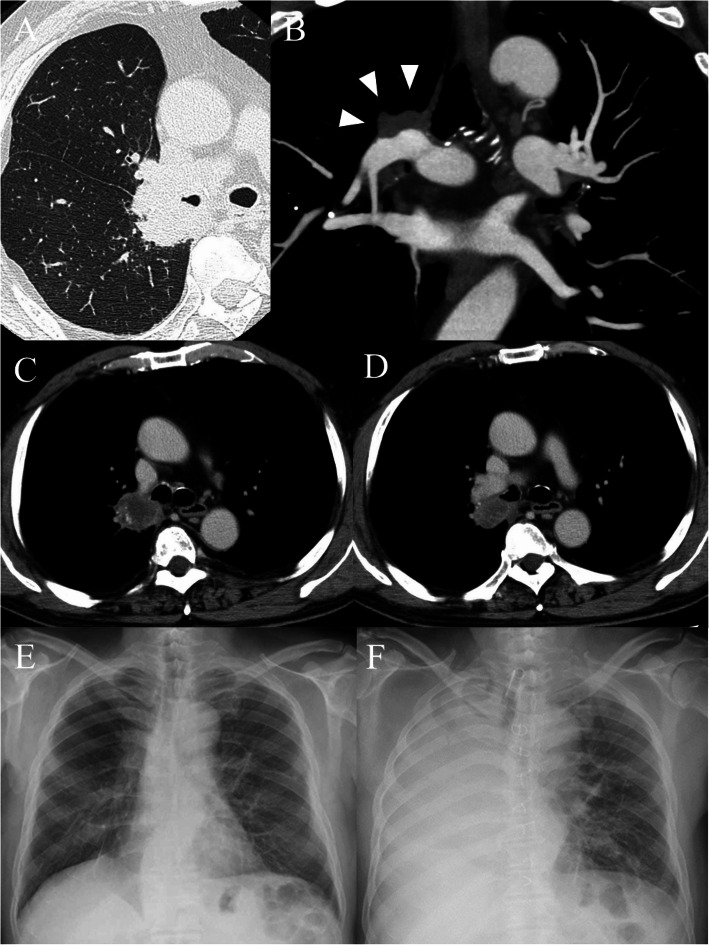
Chest computed tomography showing a tumor expanding over the remaining two lobes (**a**), compressing the pulmonary artery at the cut end of the superior trunk (**b**, arrowhead), adjoining to the azygos vein stump branching from the superior vena cava (**b**), and adhering to the membranous side of the right main bronchus (**d**). Compared with preoperative chest X-P (**e**), radiolucency was declining in the right upper lung field after pneumonia (**f**)

A 71-year-old Asian male lung cancer patient, with total obstruction of the right upper bronchus, underwent right upper sleeve lobectomy with bronchoplasty and lymph node dissection combined with preoperative induction and postoperative adjuvant chemotherapy with docetaxel and platinum agents in 2007. Thereafter, partial resection with right radical neck dissection was performed for tongue cancer of pT1N1M0. In 2016, a new undiagnosed hilar mass near the anastomosis site, as a lung metastasis of tongue cancer found on chest computed tomography (CT), was treated with radiotherapy of 36 Gy at a previous hospital. The tumor responded transiently to radiotherapy, but progressed. On chest CT at the first visit in 2018, a tumor involving the remaining two lobes compressed the pulmonary artery at the cut end of the superior trunk (Fig. 1a, b). Moreover, the tumor extended from the periphery of the anastomosis site to the tracheal bifurcation and to the inflow to the superior vena cava (SVC) of the azygos vein (ligated previously), which was ligated previously, along with the membranous side of the right main bronchus (Fig. 1c, d). In 2016, chest CT on initial detection of recurrence suggested lobar lymph node recurrence near the anastomotic site. On bronchoscopy, the bronchial anastomosis site was on the peripheral two rings from the tracheal bifurcation; tumor infiltration was suspected in the immediate periphery on that membranous side, but histological diagnosis could not be obtained from biopsy on this site. Distant metastasis was not observed in fluorodeoxyglucose-positron emission tomography, and the clinical stage was stage IB of cT2aN0M0, if it was a primary lung cancer. The high-risk patient had no abnormality in the electrocardiogram and the echocardiography in spite of having a history of coronary vasospastic angina; however, both %ppo-FEV1 and %ppo-DLco were slightly less than 40% (38.0 and 37.8%, respectively) in the pulmonary function test. Because radiotherapy has already been performed by the previous hospital and appropriate drugs could not be selected as there was no information on definitive diagnosis and gene mutation, we selected the most effective surgical treatment despite being a high-risk patient. For surgical management, after securing and cutting the proximal side of the main pulmonary artery with a median sternotomy, the tumor in contact with the azygos vein stump was divided safely from the SVC, and sleeve pneumonectomy was performed under good vision via the posterior lateral thoracotomy approach.

**Fig. 2:**
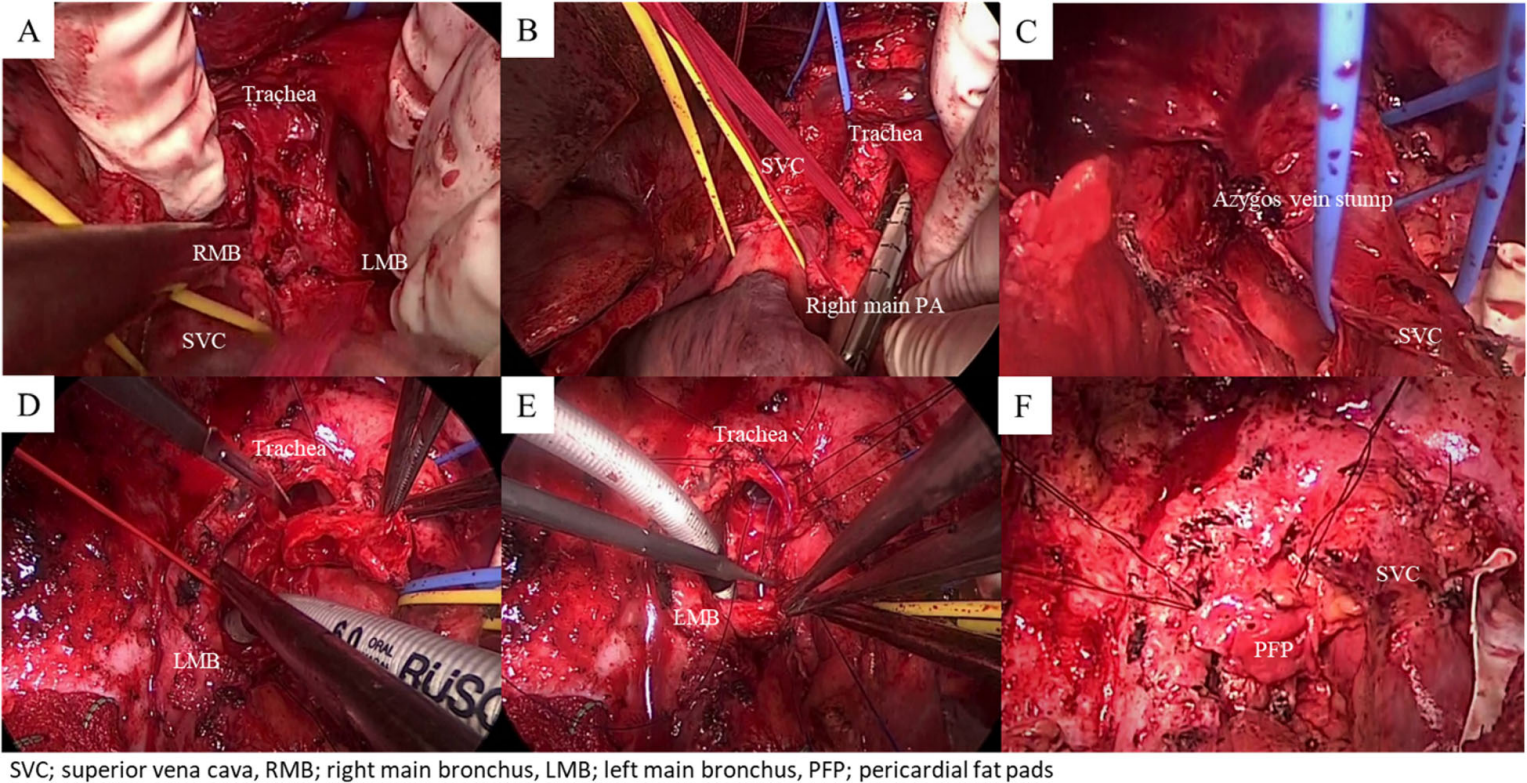
Tracheal carinal site after lymph node dissection (**a**). Incision in the right main pulmonary artery with a vascular stapler (**b**). Azygos vein stump with the main tumor after dividing at the bifurcation from the superior vena cava (SVC) (**c**). Incision in the distal trachea above the carina after intubation into the left main bronchus (**d**). Telescope anastomosis between the left main bronchus and distal trachea with interrupted sutures from both sides (**e**). Covering the anastomosis with pericardial fat pads (**f**)

Median sternotomy in the supine position was performed under general anesthesia. The proximal part of the right main pulmonary artery could only be secured in front of the left main bronchus because of severe adhesion from the tracheal bifurcation to the right main bronchus. The right main pulmonary artery was occluded for approximately 15 min to prevent deterioration of circulatory dynamics. Although the remaining lymph nodes around the trachea were dissected and the SVC was adequately detached (Fig. 2a), confirming the adhesion between the tumor and azygos vein was difficult under direct vision from this position. After dividing the proximal site of the right main pulmonary artery with a vascular stapler (Fig. 2b), posterior lateral thoracotomy was performed in the left lateral position. The azygos vein stump, which was in contact with the tumor, could be observed from the thoracic cavity side. After the inferior pulmonary vein and middle lobe vein were cut, the area around the stump of the azygos vein was peeled off and divided at the edge of the bifurcation from the SVC using vascular stapler (Fig. 2c). As the bronchial stump proximally from the anastomosis showed infiltration of cancer cells by frozen section, sleeve pneumonectomy was performed. The left main bronchus and distal trachea were exposed and mobilized with blunt dissection to avoid excessive peeling and preserve maximal blood supply. After dividing the left main bronchus, a spiral tube was intubated from the operative field; the tracheobronchial sleeve above the carina was then resected (Fig. 2d). The distal and proximal margins were confirmed radical by frozen sections. Reconstruction was performed by telescoping the left main bronchus into the distal trachea to overcome marked caliber mismatch. The distal trachea, around the anastomosis, has thickening and low-mobility area, so the first three sutures at the deepest anastomosis edge were knotted extraluminally and 15 interrupted stitches with a 3–0 PDS were placed alternately from both sides (Fig. 2e). After tying the sutures, the anastomosis site was covered with pericardial fat pads.

Postoperative pathological examination diagnosed a recurrence of lung squamous cell carcinoma because of its similarity to the previous histologic type. A tumor measuring 40 × 32 × 30 mm in diameter had central necrosis because of radiotherapy. The resected lymph nodes were free from metastasis.

Postoperatively, treatment for circulatory insufficiency associated with arrhythmia and pneumonia was required (Fig. 1f), but the patient was transferred to a public hospital on postoperative day 87 for rehabilitation. He is still alive during the 13 months after the operation without anastomotic problems and recurrence.

## Discussion and conclusions

Recently, salvage surgery is performed more frequently if strict indications are met for treatment-refractory cases or recurrence after treatment [1]. Van Schil PE presented a series of 19 completion pneumonectomy after bronchial sleeve resection involving recurrent tumor [2]. Although the survival rate was relatively good, the operative mortality rate was 15.8% because of cardiac-related disease and fistula of the bronchial stump. Sleeve pneumonectomy is extremely uncommon with 0.027–0.045% of primary lung cancer cases, and its 30-day mortality rate and hospital mortality are up to 5.9 and 29.4%, respectively [3–5]. Sleeve pneumonectomy after bronchial sleeve resection is a more invasive technique with high risk of complications and mortality than completion pneumonectomy. Therefore, it is necessary to pay attention to the following aspects of surgery: (1) surgical approach (safe vascular treatment), (2) anastomosis technique (preserving blood supply to the anastomotic site and anastomosis method), and (3) perioperative management (prevention and management for complications).

In addition to sleeve lobectomy, previous induction/adjuvant chemotherapy may cause severe inflammation and adhesion around the tracheal bifurcation. Since the main tumor was in contact with the azygos vein stump immediately after branching from the SVC, a lateral approach from the thoracic cavity was possible and safe, considering the need for SVC reconstruction.

Sleeve pneumonectomy is mostly right-sided and approached by posterolateral thoracotomy [6]. In this case, it was difficult to mobilize and divide the pulmonary artery more proximally via the lateral approach; therefore, median and posterolateral incisions or hemi-clamshell incision was considered optimal. Although this surgical approach took a longer time including postural change, complicated blood vessel treatment could be performed under a good visual field without stress.

Maximal blood supply preservation and avoiding excessive tension are important for successful anastomosis. Tracheal blood flow is mainly supplied from the esophagus side, so the detachment of the dorsal side of the distal trachea was minimized in this case. Anastomosis from both sides is necessary for effective visualization of the anastomosis site due to the thickened tracheal wall and surrounding tissues and decreased mobility associated with adhesion. We used preferentially interrupted suture for cases with marked caliber mismatch or thickened bronchial wall. In this case, there was no caliber mismatch; therefore, the anastomosis was performed with an interrupted suture without adjusting the anastomosis. Telescope anastomosis easily corrects massive caliber mismatch by intussusception of the left main bronchus into the distal trachea and prevents kinking and excessive tension [7]. In the present case, no obvious anastomosis-related complications were noted during follow-up.

Postoperative anastomotic complications occur in up to 14% of sleeve pneumonectomy cases, with mortality rate up to approximately 50% with a poor prognosis [8]. Although anastomotic procedures are associated with anastomotic complications, preoperative treatment is important. In preoperative radiation therapy, high-dose irradiation increases the risk of bronchopulmonary fistula, and sleeve pneumonectomy has been reported as a relative-absolute contraindication for dose > 45 Gy [6]. The irradiation dose in our case was 36 Gy, and no anastomotic complication occurred.

In conclusion, salvage surgery is performed more frequently if strict indications are met for treatment of refractory or recurrent cases. For airway reconstruction surgery on the proximal side, the difficulty of securing the airway and vessels more centrally and the effect of prior treatment, such as radiotherapy and/or chemotherapy, make the operation more difficult. The two-stage approach provides a good field of view and operative field and can be safely performed even in difficult operations.

## Data Availability

All data generated or analyzed during this study are included in this published article.

## Abbreviations

CT: Computed tomography
SVC: Superior vena cava
